# Degradation Products of Tryptophan in Cell Culture Media: Contribution to Color and Toxicity

**DOI:** 10.3390/ijms22126221

**Published:** 2021-06-09

**Authors:** Alisa Schnellbaecher, Anton Lindig, Maxime Le Mignon, Tim Hofmann, Brit Pardon, Stephanie Bellmaine, Aline Zimmer

**Affiliations:** Upstream R&D, Merck KGaA, Frankfurter Strasse 250, 64293 Darmstadt, Germany; alisa.schnellbaecher@merckgroup.com (A.S.); anton.lindig@tu-dortmund.de (A.L.); maxime.le-mignon@merckgroup.com (M.L.M.); tim.hofmann@merckgroup.com (T.H.); brit.pardon@merckgroup.com (B.P.); aline.zimmer@merckgroup.com (A.Z.)

**Keywords:** tryptophan, color, cell culture media, LC-MS, antioxidant, cytotoxicity, biomanufacturing

## Abstract

Biomanufacturing processes may be optimized by storing cell culture media at room temperature, but this is currently limited by their instability and change in color upon long-term storage. This study demonstrates that one of the critical contributing factors toward media browning is tryptophan. LC-MS technology was utilized to identify tryptophan degradation products, which are likely formed primarily from oxidation reactions. Several of the identified compounds were shown to contribute significantly to color in solutions but also to exhibit toxicity against CHO cells. A cell-culture-compatible antioxidant, a-ketoglutaric acid, was found to be an efficient cell culture media additive for stabilizing components against degradation, inhibiting the browning of media formulations, and decreasing ammonia production, thus providing a viable method for developing room-temperature stable cell culture media.

## 1. Introduction

One strategy for improving biopharmaceutical manufacturing processes is to optimize cell culture media (CCM). The latest drive in the industry is to improve the chemical stability of hydrated media against degrading conditions such as exposure to light and increased temperatures. Detrimental light exposure can be encountered unintentionally in the transport and storage of CCM or during the cell culture itself, as modern cell culture bioprocesses are often carried out in reaction vessels that are exposed to light [1,2]. It is standard practice to maintain CCM at 4 °C prior to use, but the design of hydrated CCM that are stable at room temperature (RT) may decrease the energy consumption by eliminating the need for refrigeration. This optimization of CCM stability involves both developing methods to detect critical changes to CCM that can negatively impact cell culture [3,4] and using this information to discover strategies to mitigate such changes.

When exposed to stressed conditions (e.g., light, heat), sensitive CCM components decompose and new degradation products are generated. This chemical change in the media composition can often be directly observed by a change in the color of the media. This is a greater issue for feeds of a fed-batch process than for basal media, due to the higher concentration of components. This color change is not only a warning signal for a chemically altered CCM but is of added concern for the potential to alter the color of the drug product, a critical quality attribute (cQA) of therapeutic monoclonal antibodies (mAbs). The ability of degraded CCM components to alter the color of the mAb product was best demonstrated by Prentice et al., who showed that the light-induced degradation of cyanocobalamin (vitamin B12) to hydroxocobalamin caused the mAb product to become pink-colored [5]. However, while many reports explore the contributions of particular CCM components and specific degrading conditions to mAb coloration [5,6,7,8], researchers have yet to explore the coloration of CCM itself. The color change associated with the degradation of some cell culture media components has been reported, such as for the Maillard reaction of amino acids (AAs) and glucose, which leads to highly oxidized, highly colored organic compounds [9,10,11]. Other common organic CCM components shown to produce colored degradation products are thiamine [12,13] and tryptophan [14,15]. The instability of CCM components is not only an issue for color production, but also for the potential production of toxic degradation products, which would be detrimental to cell culture. Methods to inhibit color change in CCM when exposed to stressed conditions (e.g., heat, light) may also then mitigate the production of toxic degradation products, which would be an additional benefit for the biomanufacturing industry.

In this study, the major organic compounds (i.e., amino acids and vitamins) in a feed, developed for CHO-based bioproduction, were investigated for their contribution to coloration (also known as browning) under stressed conditions, with a focus on attaining knowledge that may be used to develop RT stable CCM. Tryptophan (Trp) was found to be the major contributor. LC-MS technology was used to identify tryptophan degradation products which were built up in the stressed conditions that correlated with browning, and these products were tested for their ability to contribute to feed coloration and cellular toxicity. An antioxidant known to be tolerated in high concentrations in cell culture was investigated for its ability to reduce browning and/or the generation of detrimental Trp degradation products. This work contributes heavily to the understanding of Trp degradation in cell culture media and other aqueous solutions, including advanced knowledge of Trp degradation products and methods toward inhibiting problematic degradation, especially the generation of toxic or highly colored species.

## 2. Results

### 2.1. Tryptophan Is a Major Inducer of Browning in a Feed Used for CHO-Based Bioproduction

**Figure 1:**
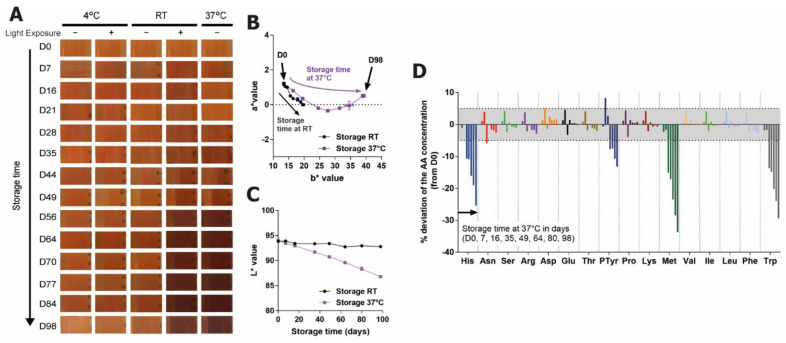
Discoloration and amino acid degradation of feed upon 98 days of storage in stressed conditions: (**A**) images of feed throughout the stability study; changes in (**B**) a* and b* values and (**C**) L* values of the RT and 37 °C stored samples (no light exposure); (**D**) changes in amino acid content of feed upon 37 °C storage (no light exposure). Gray zones indicate the maximum variability due to the analytic method.

Experiments investigating the effects of increased temperature and light exposure in the long-term storage of feed showed no color change at 4 °C storage, but storage at RT in the presence of light or at 37 °C in the absence of light both caused a stark browning of the medium (Figure 1A).

A more precise measure of the color change was determined using the CIE L*a*b* color system (See Supplementary Materials Section S1 for details) [16,17]. In the sample stored at 37 °C, the b* value shifted consistently in a positive direction over time (becoming more yellow), while the a* value decreased slightly at first (became more green) and then increased again (more red). The RT sample did not exhibit such a strong color shift in these axes but followed the same trajectory as the 37 °C sample (Figure 1B). The RT sample showed no change in L*, while for the 37 °C sample, the L* value decreased notably, which is representative of the darkening of the solution over time (Figure 1C). The similar a*b* trajectory in the two samples but stronger L* changes in the 37 °C sample may indicate that the increased temperature is causing the same degradation, but at a faster rate. Investigations into the changes in the amino acid levels of these two solutions showed that at RT, only methionine (Met) and Trp were significantly affected (data not shown), while at 37 °C histidine (His) and phosphotyrosine (PTyr) were also degraded (Figure 1D). The PTyr decrease at 37 °C is known to be the result of PTyr being converted to Tyr, as opposed to being degraded to other components [18]. The vitamins were also investigated for their contribution to color, but this was found to be negligible (data not shown).

**Figure 2:**
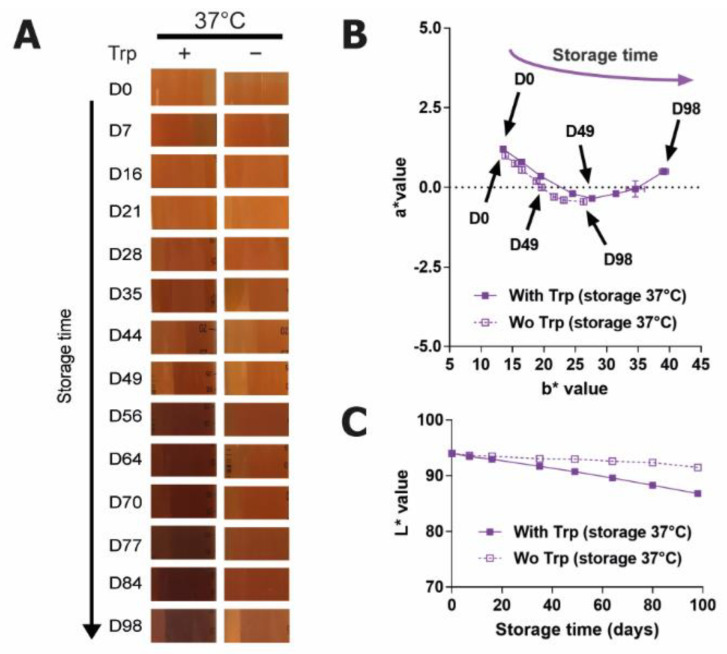
Stability study of the feed with or without (wo) Trp at 37 °C over 98 days. (**A**) Images of the solutions. (**B**) Changes in a*/b* and (**C**) L* values in the solutions.

Due to the considerable amount of literature regarding the production of yellow degradation products from tryptophan [8,19,20], initial experiments focused on the possible contribution of Trp to the color of the degraded feed, by comparing the complete feed to a version lacking Trp. The presence of Trp in the feed caused a clearly visible increase in browning (Figure 2A). The increase in b* with Trp inclusion suggested a greater increase of color in the yellow coordinate (Figure 2B), while the decrease in L* indicated a much stronger darkening of color over time (Figure 2C). This shows that there is a significant color contribution to the solutions as a result of the presence of Trp. The degradation profile of the sensitive AAs His, Met, and PTyr were all comparable in both solutions (data not shown), demonstrating that the degradation of these AAs has no bearing on color change in the solution. The degradation of these AAs even in the absence of Trp indicates that Trp degradation is not inducing the degradation of any of the other AAs, and vice-versa. These experiments confirm that Trp is the major component producing brown products in this feed.

### 2.2. Identification of Degradation Products from Trp in Water Stored under Stressed Conditions 

**Figure 3:**
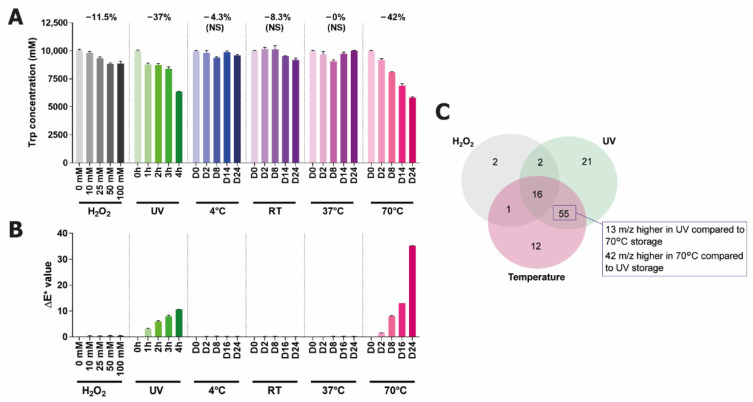
Degradation of tryptophan in water (10 mM, pH 7) under stressed conditions. The conditions tested were stored with varying concentrations of hydrogen peroxide, exposure to UV light, and storage at varying temperatures for 24 days. (**A**) Changes in Trp concentration (NS, non significant). (**B**) Color change displayed as ΔE*. (**C**) Venn diagram of the features identified in LC-MS above an abundance threshold of 30,000.

Having established that Trp degradation is the lead cause of browning in this feed, the next step in understanding the color change was to determine which degradation products are being generated. Due to the complexity of the solutions, LC-MS was chosen as the optimal method for identifying degradation products of interest. The method was first applied to degraded solutions of Trp in water, considering this might also help identify components in the feed that are coming exclusively from Trp self-reactions. Additional conditions also known to degrade Trp were included in this part of the study, namely exposure to hydrogen peroxide, exposure to UV light, and storage at various temperatures [14]. This experiment was shorter in duration (24 days) but included a 70 °C stressed condition, as representative of an accelerated degradation condition. The measurement of the Trp concentration changes showed that the UV light exposure and 70 °C conditions resulted in the greatest Trp degradation (37% and 42% total loss after 24 days, respectively), followed by hydrogen peroxide incubation, while the storage at 4 °C, RT, and 37 °C induced minimal Trp degradation (Figure 3A). Changes in the parameter ΔE*, which broadly quantifies the overall color change in the L*a*b* system (see Supplementary Materials Section S1) [17] showed that the 70 °C condition changed color most significantly, followed by UV, while the other solutions did not change at all (Figure 3B). For the temperature and UV conditions, there is clearly a correlation between the extent of Trp degradation and the increase in color. Interestingly, the degradation caused by hydrogen peroxide did not lead to a correlating increase in color.

**Figure 4:**
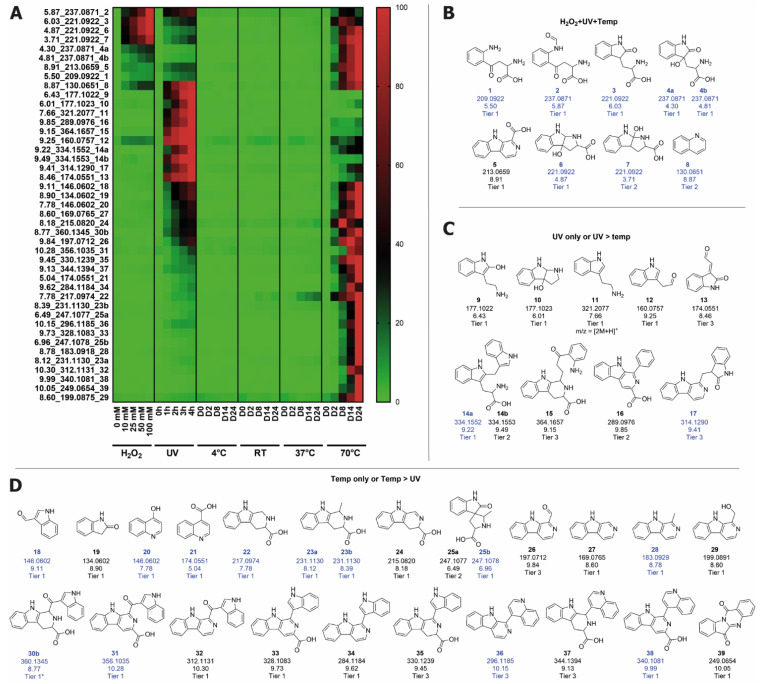
Tier 1–3 features in the aqueous Trp samples which were significantly modulated in at least one of the stress conditions. (**A**) Normalized abundance profiles of each feature (rt_m/z_structure number), presented in a heatmap. Green represents the lowest abundance and red the highest abundance for each feature independently. Structures for features that were increased (**B**) in all stress conditions, (**C**) in UV only or UV > 70 °C conditions, and (**D**) in 70 °C only or 70 °C > UV conditions. All m/z represent [M+H]^+^ unless specified otherwise (shown with structures). Features that are also present in the 37 °C feed experiment (Table S1) have the compound number and feature data displayed in blue.

With the knowledge of which stress conditions result in an increase in color, LC-MS was employed to determine the major degradation products in each condition, and ultimately which products were contributing to browning. LC-MS features that corresponded to unique compounds were characterized with unique identifying information (retention time (rt) and *m*/*z* for a given parent ion) and focus was given to analyzing the most prominent features, defined as those generated above a chosen abundance threshold (>30,000) (see Supplementary Materials Section S2 for detailed data processing). Fifty-five features were found to be increased in both the UV and the 70 °C conditions, i.e., the conditions in which browning occurred. This indicates that these features are those most likely to represent small molecules that are contributing to browning. In addition, 42 of these features presented an abundance significantly higher in the 70 °C compared to the UV condition, corresponding to the relative magnitude of browning in these conditions, and as such the small molecules corresponding to these features may be those most significantly contributing to browning. Structure suggestions for these features were made based on literature knowledge or by the analysis of the MSMS fragmentation pattern. Where possible, standards were purchased or synthesized to attain a confirmation for the proposed structure (see Supplementary Materials Figure S1 for an example of matching MSMS profiles for standard and experimental data). The structure proposals for each feature were assigned a confidence level (a tier) based on best practices for small molecule structure identification using LC-MS [21,22]. A compound was assigned tier 1 when the MSMS data and rt matched with a pure standard and tier 1* if the confirmation was via a standard that could not be purified, but for which the structure assignment based on the synthetic route was supported by the literature (see Supplementary Materials Section S2 for detailed tier descriptions). Of the 109 features detected as significantly modulated in these aqueous solutions of Trp, 30 features were assigned to tier 1 or 1*, corresponding to a total of 28 confirmed compound structures, as some features were assigned as diastereomers (absolute configuration unknown—features with earlier rt are designated as ‘a’ and later rt as ‘b’) (Figure 4).

Five of these compounds were found in all degradation conditions and correspond to major known degradation products of Trp: kynurenine (KYN, **1**), *N*-formylkynurenine (NFK, **2**), oxindolylalanine (Oia, **3**), dioxindolylalanine (diOia, **4a** and **4b**), and pyrolloindole-3-carboxylic acid (PIC, **6**) [23]. Within the tier 1 features that increased in abundance in the UV and/or 70 °C condition and may therefore be contributing to browning, three main structure classes were identified—b-carbolines, quinolines, and indoles. It is well established that tetrahydro-b-carboline structures can be easily formed from Trp via a condensation reaction with aldehydes and ketones known as the Pictet-Spengler reaction [24], and that these structures can readily undergo subsequent oxidation, optionally with concomitant decarboxylation [25]. This pathway easily explains the presence of the many b-carboline products, and in some cases the direct aldehyde precursor was also detected, as is the case for aldehyde **18**, which is potentially a direct precursor for carbolines **33** and **34**. Quinolines have been shown to be formed from Trp via the initial formation of KYN (**1**) [26], which provides a sensible explanation for the appearance of compounds **8**, **20**, and **21**. While the indole moiety in the products is carried over from Trp, most indole structures exhibit additional oxidation (e.g., **9**, **19**, **25b**). High levels of oxidation are prevalent in most of the degradation products, especially in the products formed at higher temperatures, such as the fully unsaturated b-carbolines (e.g., **26**–**29**, **31**–**34**, **36**, **38**). The oxidation of tetrahydro-b-carbolines by atmospheric dissolved oxygen has been reported [25,27]. The presence of spiro[pyrrolidineoxindole] **25** also indicates that the storage conditions are highly oxidizing. This spiro compound may be formed either via a Mannich reaction (another type of condensation reaction) between acetaldehyde and the oxidation product Oia (**3**) or from the oxidative intramolecular rearrangement of the tetrahydro-b-carboline **23** [28]. Tryptanthrin (**39**) is a natural product produced by yeast and plant species. Despite being a natural product, many methods have been developed to synthesize this structure chemically, usually involving the oxidation of an indole-containing precursor such as indole or isatin, which indicates that this compound is also probably formed in these samples via oxidative reactions [29]. The only products exhibiting signs of reduction formed predominantly in the UV condition and are likely caused by radical/photochemical reactions, as has been shown in the literature for compounds **11** and **14** [30,31]. Structures were also proposed for 13 other features, falling into the tier 2 or 3 categories (Figure 4). These contain the same functional groups as seen in the tier 1 compounds and therefore the generation of these compounds in aqueous solutions under these degradation conditions is highly likely. The remaining features in this experiment fell into the tier 4 and 5 categories, and their corresponding abundance data are located in Supplementary Materials, Figure S2.

### 2.3. Identification of Degradation Products Originating from Trp in Feed Stored at Elevated Temperature

**Figure 5:**
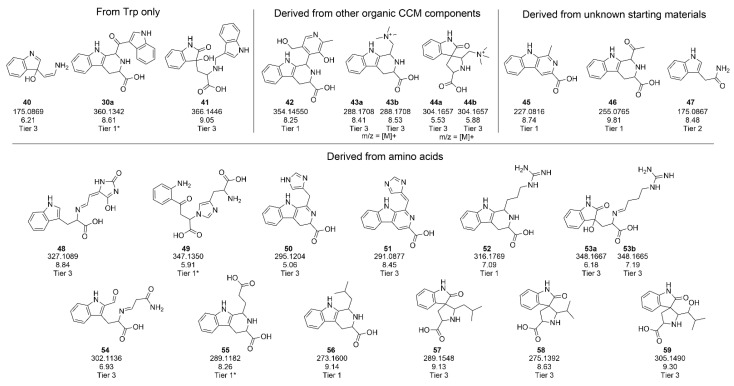
Structures for novel Trp degradants detected in feed studies (those features detected in both water and feed studies are shown in Figure 4 with their feature data marked in blue). Structures are provided for tier 1–3 features that were significantly modulated in the Trp-containing feed compared to the Trp-depleted feed. All *m/z* represent the parent peaks detected as [M + H]^+^ ions, unless indicated otherwise.

While the industry is predominantly interested in developing RT stable feed, for the purposes of detecting degradation products, 37 °C was chosen as representative of an accelerated degradation condition. An LC-MS analysis of the degraded solutions previously analyzed for color (Figure 2A) identified 85 features that were highly abundant only in the feed containing Trp (Table S1). Of the 47 features that were categorized into tiers 1–3 (55% annotation of features), 22 features were also found in the aqueous Trp samples (corresponding features have the compound number and feature data in blue in Figure 4 and are indicated with an asterisk next to the compound number in Table S1). Additional stability studies of feeds selectively depleted in other organic CCM components were used to establish the origins of some of the features (CCM component origin shown in Table S1). Some features were identified as having arisen solely from Trp from the water-only study (e.g., **40**, **41**) (Figure 5). A total of 25 features were clearly derived from AAs, and 13 of these were classified as tier 1–3. Seven features were derived from non-AA organic CCM components (namely choline, thiamine or pyridoxine) and five of these were categorized into tiers 1–3 (**42** from pyridoxine and **43a/b** and **44a/b** from choline). As with the water solutions of Trp, the structures of tier 1 features in feed contain many of the same functional groups (e.g., ketone, imine, alkene) and structural skeletons (e.g., b-carboline, spirane), indicating that the same kinds of degradative chemical processes (e.g., condensation, oxidation) occur in feed and water samples. Sixty-seven features (39 in tiers 1–3) increased in abundance over time and thus correlated with the increase in browning (Table S1). The compounds correlating with browning show many common features—a high level of oxidation and almost always the presence of an aromatic ring. It is well understood that increasing aromaticity and conjugation in an organic compound leads to a greater ability to absorb light [16,17], so it is possible that these compounds are contributing directly to the color increase. However, an examination of the direct contribution of these compounds to color in solution is required to make such a claim. It is important to note that features that were prominent in the water study but not in the feed study are not necessarily absent from the feed. The same features may be generated in feed below the cutoff threshold chosen for data analysis or may be difficult to detect in the complex matrix due to signal suppression.

### 2.4. Color Contribution of Tier 1 Compounds to Water and Feed Solutions

**Figure 6:**
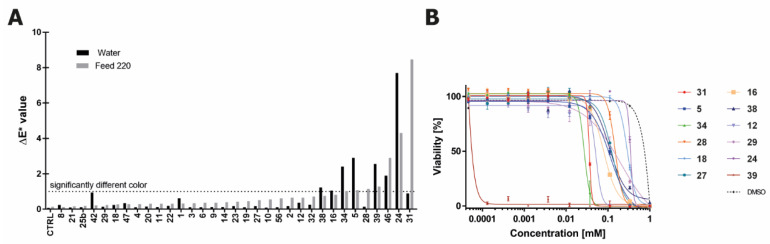
(**A**) Color change as measured by ΔE* in both water and feed upon addition of degradation products. Solutions of degradation products in DMSO were spiked into aqueous solutions to yield a theoretical concentration of 500 µM (1% DMSO). (**B**) Toxicity data for the Trp degradation products that produced a significant toxicity in CHOK1 GS cells as compared to the DMSO control. Error bars, S.D.

Standards available in significant enough quantities were used for spiking experiments to see whether they contributed to the coloration of either pure water or feed. ΔE* > 1 was deemed as a significant change (Figure 6A). Some of these compounds have already been identified as possessing notable color. Tryptanthrin (**39**) and pityriacitrin (**32**) are both natural products characterized as yellow solids [29,32], and pityriacitrin was shown to have broad absorption in the UV spectrum [32].

Compounds were dissolved in DMSO at 50 mM and then spiked into the solutions to achieve a final compound concentration of 500µM. Some of the tested compounds formed a precipitate immediately upon spiking of the DMSO solution. As the precipitates in solution can interfere with the absorption measurement, each sample was centrifuged prior to taking absorption measurements, meaning that the final concentrations were not all at 500 µM. This interference from precipitation was shown not to occur in the complete feed samples, as the L*a*b* measurements in the 37 °C samples at 98 days of storage were shown to be identical before and after the filtration of the medium through a 0.22 µm filter (data not shown). A significant color change was produced by compounds **16** and **38** in water, by compounds **28** and **31** in feed, and by compounds **5**, **24**, **34**, **39**, and **46** in both conditions (Figure 6A). In stability experiments, eight of these nine compounds (all except **46**) were present in at least one condition in the degraded water samples, and four of these nine compounds (**28**, **31**, **38**, and **46**) were present in the degraded feed. Compounds **28**, **31**, and **46** all had significant abundances in the degraded feed samples and displayed a significant production of color in the feed, which would suggest that these compounds may be critical to enhance color in feed stored at enhanced temperatures.

### 2.5. Toxicity of Trp-Derived Degradation Products in CHO Cells

Four of the established Trp degradants are natural products and have been tested previously for various types of cellular toxicity. Pityriacitrin (**32**) and pityriacitrin B (**31**) have demonstrated toxicity against some cancer cell lines [33], and while eudistomin U (**34**) displayed some toxicity in human cancer cell lines, it was most potent as an antibacterial agent [34]. Tryptanthrin (**39**) is known to exhibit low micromolar toxicity against a wide range of both cancerous and non-cancerous human cell lines [29,35,36]. As we have demonstrated that these compounds are generated from degraded Trp, it was of interest to investigate the toxicity of these and the other established Trp degradants in CHO cells, the most prevalent cell type used in the biomanufacturing industry. The same panel of standards previously tested for color effects were tested for toxicity in a CHOK1 GS cell line. Twelve compounds were found to elicit notable toxicity in this cell line (GI_50_ < 1 mM) (Figure 6B) and were therefore tested for toxicity in three other CHO cell clones. The compounds showed roughly the same toxicity trends across the four cell lines (see Supplementary Materials, Table S2). Tryptanthrin (**39**) displayed extremely high toxicity across all cell lines (GI_50_ ≤ 1.1 µM), while eudistomin U (**34**) and pityriacitrin B (**31**) were fairly toxic (10 < GI_50_ < 22 µM across all cell lines) and compounds **18** and **24** displayed the lowest toxicity of the twelve (GI_50_ > 226 µM).

### 2.6. Inhibition of Browning and Degradation Product Formation Using Alpha-Ketoglutaric Acid 

**Figure 7:**
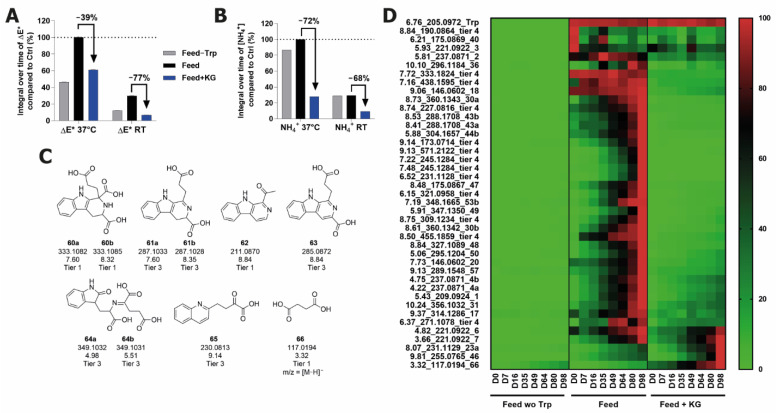
Changes in the feed, feed-Trp, and feed+aKG solutions during long-term storage. (**A**) Color change measured as ΔE*. (**B**) Changes in ammonia concentration. (**C**) Structure identifications for tier 1–3 features which are newly formed upon aKG addition to feed. (**D**) Heatmap showing abundance changes of features of interest (denoted with rt_m/z_structure number for tiers 1–3, or else denoted as tier 4) at RT.

With the observation that the structures correlating with increased browning showed distinct characteristics of oxidation (incorporation of oxygen atoms, increase in aromaticity), the application of a known antioxidant as a method to reduce the browning and concomitant production of these degradants was tested. a-Ketoglutaric acid is a common CCM component with antioxidative properties, which has been shown to have no detrimental effect on the CHO cell culture up to 50 mM and to protect against the temperature-induced degradation of some organic CCM components [37]. The ketoacid was tested for its ability to minimize browning and the production of Trp degradation products in feed stored at RT or 37 °C for 98 days. The ammonia levels in the solutions were also measured, as this could arise from several of the identified Trp degradation products in which nitrogen loss is observed (e.g., **12**, **18**, **19**). Increased ammonia in CCM is undesirable, as it can adversely affect cell growth and product quality [38,39,40]. The addition of aKG induced a 39% decrease in browning for feed stored at 37 °C, and a 77% decrease at RT (Figure 7A). This demonstrates that the addition of aKG is highly effective in decreasing browning, as the color change at RT was lower in the +aKG condition than in the no Trp control.

In both conditions, the decrease in ammonia (72% at 37 °C, 68% at RT) far exceeded the decrease brought about when Trp was excluded (13% at 37 °C and 0% at RT) (Figure 7B). This suggests that the ammonia production in the feed is not coming exclusively from Trp, and that aKG is also inhibiting the ammonia-generating degradation of other organic CCM compounds, most probably other AAs, as AA deamination at higher temperatures is a well-established cause of ammonia production [41].

In the 37 °C condition, 62 features in total (see Supplementary Materials Figures S3 and S4) decreased in abundance upon aKG supplementation, five features exhibited similar abundance profiles, and the formation of nine features was inhibited completely. However, 12 features had increased abundances with the addition of aKG, and another 14 features were detected only in the feed+aKG condition. Some of the newly formed features were identified as compounds formed from the combination of Trp and aKG, e.g., tetrahydro-b-carboline **60** and its oxidative decarboxylation product, **61** (Figure 7C). For aKG addition at RT storage, none of these new Trp+aKG features were detected, and 36 features were markedly reduced or eliminated completely (Figure 7D). Only one remained at a comparable abundance with the control (tier 2, corresponding to pyrroloindole **7**), and only two features had increased abundance (tier 1, corresponding to structures **23** and **46**), which were both also increased at 37 °C. Many of the features that had lower abundances in the +aKG condition were those shown to derive from other CCM components (e.g., **43**, **44**, **49**, **53**, **57**). Many features corresponding to structures with high oxidation levels were decreased down to levels comparable to the no Trp control (e.g., **1**, **4**, **18**, **20**, **30**, **36**). The decreased abundance of these Trp products combined with the reduction in browning and ammonia production to below the no Trp control level suggests that aKG may be protecting multiple CCM components from degradation via an antioxidant function. Not much is known about the function of aKG as an antioxidant, although it is known that the reaction with the oxidant hydrogen peroxide causes the oxidative decarboxylation of the a-ketoacid, forming succinic acid (**66**) [42]. The feature corresponding to this compound was detected in the feed in negative mode (Figure 7D), and the abundance was increased in the feed+aKG condition, supporting the hypothesis that aKG is functioning as an antioxidant. It is curious to note that the Trp degradation was not decreased by aKG supplementation, but a complete understanding of the chemical changes induced by aKG addition is beyond the scope of this study.

## 3. Discussion

This study demonstrated that tryptophan was the major contributor to the browning of a selected feed. The identification of several Trp degradation products demonstrated that the condensation reactions of CCM components and oxidation reactions were the major chemical processes taking place at elevated temperatures. These combinations of reactions often yielded products with fully oxidized heterocycles and a high level of conjugation, which are typical properties in colored organic compounds, and correlates with the color increase in the solutions. As these compounds are significantly different in structure from Trp and as protein translation involves many quality control systems to avoid the incorporation of incorrect AAs [43], it was deemed unlikely that the incorporation of these degradation products in the drug product would be a concern. However, the discovery that some of these compounds can contribute directly to CHO toxicity and feed browning indicates that these degradation processes can be detrimental in biomanufacturing, and therefore methods need to be established to limit such degradation in CCM that are intended to be stored at elevated temperatures. The submicromolar toxicity of tryptanthrin (**39**) is especially noteworthy. Its chemical structure is significantly different from that of all of the other compounds tested (it is the only compound with a indolo[2,1-*b*]quinazoline core), and is therefore likely the fundamental reason behind its uniquely high toxicity. It is not clear by which mechanism tryptanthrin achieves such high toxicity in CHO cells, but in other cellular toxicity studies it commonly bound to ATP-binding sites of crucial enzymes in pathways involved in apoptosis regulation, cellular proliferation, and cell survival [25]. Tryptanthrin could therefore be eliciting toxicity via a similar means in CHO cells. For studies such as this one, which investigate the effects of organic compounds on cell culture, it is relevant to note that very small amounts of transition metal contaminants can be the cause of the observed changes (e.g., color and toxicity) [44,45,46], and as such follow-up studies may be required to confirm that any detrimental effects are indeed due to the organic component, though this is currently beyond the scope of this work. The addition of a-ketoglutaric acid to the cell culture feed was shown to decrease the detrimental chemical changes in the feed stored at higher temperatures, including browning, ammonia production, and the generation of Trp-derived degradation products. The extent of ammonia reduction suggests that aKG is inhibiting the deamination of other AAs, and the decrease in major Trp oxidation products, along with the production of succinic acid, suggest that this may result from aKG functioning as an antioxidant. The production of succinic acid as a byproduct is possibly even advantageous, as succinic acid is not only colorless in solution and not toxic to CHO cells, but has even shown potential benefits for mAb production in CHO cell cultures [47,48]. This work demonstrates the first known application of an antioxidant to stabilize CCM stored at higher temperatures, and thus represents perhaps the beginning of the next major CCM optimization strategy for the bioprocessing industry.

## 4. Materials and Methods

### 4.1. Reagents

Raw materials and cell culture media/feed in the stability studies were all purchased from Merck KGaA, Darmstadt, Germany, if not stated otherwise. Chemical standards for LC-MS feature identification were purchased from a commercial supplier and synthesized externally under contract or in-house. Details of the origins of the standards are provided in the Supplementary Materials in Table S3. The feed formulation used in this study was a customized dry powder derived from Cellvento^®^ Feed220 (Merck, Darmstadt, Germany), depleted in amino acids, and was reconstituted according to the manufacturer’s guidelines, with amino acids added back as desired.

### 4.2. Stability Studies

For the Trp solutions, 10 mM Trp solutions were prepared in water with a pH adjustment to pH 7.0 ± 0.3 using NaOH(aq.) and sterile filtered with Steriflip^®^ filter units (Merck, Darmstadt, Germany). For H_2_O_2_ exposure testing, the solutions were spiked with hydrogen peroxide (30%, 8.82 M) to 0, 10, 25, 50, or 100 mM, and stored for 4h at RT (~19–23 °C), light-protected. For the UV exposure effects, the solutions were stored for 4 h under a UV-lamp set to an intensity of 540 V (UV-A, -B and -C radiation). Samples tested for temperature effects were stored light-protected for 24 days at 4 °C, room temperature (RT, i.e., ~19–23 °C), 37 °C, or 70 °C. Feed solutions were sterile-filtered as above and stored for 98 days at 4 °C, RT (~19–23 °C) or 37 °C. The samples were either stored light-protected or exposed to laboratory fluorescent lighting with partial exposure to glass-filtered daylight (light-exposed). For testing the a-ketoglutaric acid effects in feed, dry powder feed was reconstituted with a-ketoglutaric acid, pH adjusted to 7.0 ± 0.3 with NaOH (aq.), filtered as above, and stored light-protected at RT (~19–23 °C) or 37 °C for 98 days. Ammonia was quantified using Cedex Bio HT (Roche, Mannheim, Germany). Aliquots were frozen at −20 °C for subsequent amino acid analysis and LC-MS.

### 4.3. CIELAB Color Analysis

Absorbance measurements were taken in the range of 380 nm to 780 nm (20 nm steps) of 100 µL of the samples in four technical replicates (Envision, Perkin Elmer, Waltham, MA, USA). From the absorbance values, the change in color was calculated using L*a*b* and ΔE* values according to DIN 5033-3 and DIN 6174 norms. The CIE 1931 standard observer (2°) and the D65 illuminant were selected.

### 4.4. Amino Acid Analysis

Amino acid analysis was performed using a pre-column derivatization using the AccQ Tag^®^ Ultra Reagent kit. Derivatization, chromatography, and data analysis were performed according to the supplier recommendations (Waters, Milford, MA, USA).

### 4.5. LC-MS Feature Determination and Structure Elucidation

Time point series from stability study experiments were analyzed using UHPLC (Vanquish, Thermo Fisher, Waltham, MA, USA) coupled with an ESI-Q-ToF mass spectrometer (Impact II, Bruker, Bremen, Germany). Briefly, the samples were diluted 10 times in water prior to LC-MS/MS analysis. Five microliters of sample were loaded in 99.9% buffer A (20 mM ammonium formate/0.1% FA) onto a XSelect HSS T3 column (2.1 × 150 mm, 3.5 µm, Waters, Milford, MA, USA) thermostated at 40 °C with a flowrate of 300 µL/min. An optimized 12 min linear gradient was applied using 100% methanol (buffer B) as follows (minute/% B): 0/0.1, 2/0.1, 4/20, 6/30, 8/80, 8.5/100, 9.5/100, 9.6/0.1, 12/0.1.

LC-MS/MS analyses were performed in triplicate using the Impact II mass spectrometer equipped with an ESI source (Bruker, Bremen, Germany). MS acquisition was performed in positive and negative modes with capillary voltages set at 4500 V and 3500 V, respectively, and the end plate offset set at 500 V. Nebulizer and dry gas (250 °C) were set at 1.4 bar and 9.0 L/min, respectively. MS spectra were acquired over the m/z range 20–1000 with a scan rate of 12 Hz followed by data-dependent auto-MS/MS acquisitions using a fixed MS-MSMS cycle time of 0.5 s and a summation time adjusted to the precursor intensity. The data analysis was performed using Data Analysis 4.0 (Bruker, Bremen, Germany) and Progenesis QI (Non-linear Dynamics—Waters, Newcastle, UK). Briefly, raw data were processed as centroid data using the automatic peak detection algorithm of Progenesis QI. No normalization was applied to the dataset due to the nature of the study. Unique ions (retention time (rt) and *m/z* pairs) were grouped, and their abundancies were summed to generate unique features. Only features satisfying the following criteria were considered: abundance >30,000; fold change >3; corrected ANOVA *q*-value < 0.05. Hierarchical clustering was used to identify groups (e.g., identify all Trp related features by using a feed depleted in Trp and selecting the features that are not found in that condition). For the representation in the heatmap, the data were normalized between the minimum and the maximum abundance for each replicate independently. Feature annotations were performed within Progenesis QI using precursor mass, fragment mass, and retention time (if available) with tolerances set at 5 ppm, 20 ppm, and 0.3 min, respectively. Comprehensive LC-MS data is provided in Supplementary Materials 2.

### 4.6. Color Measurements of Standards

Chemical standards were prepared as 50 mM stocks in DMSO and then spiked into the aqueous solutions (either water or feed) to achieve a theoretical compound concentration of 500 µM (1% DMSO). The solutions were centrifuged, and the absorbance measurements of these solutions was measured and converted to ΔE* values as described above. The control condition was pure DMSO (1% *v/v*).

### 4.7. Cytotoxicity Assay

To assay the potential toxicity induced in the CHO cell culture by the Trp degradation products found in this study, a cytotoxicity assay CellTiter-Glo^®^ Luminescent Cell Viability Assay (Cat #G7572, Promega, Madison, WI, USA) was used, following the manufacturer’s instructions. Briefly, the cells were seeded with 25,000 viable cells per cavity of a 96-well opaque white cell culture plate (Thermo Fisher, Waltham, MA, USA) in 80 µL of a pre-warmed medium. Tryptophan degradation compounds were added in triplicates to the cells, ranging from 1 mM to 46 nM. A DMSO control was added in the same concentration range as the tryptophan degradation compounds were diluted. The dose response curves were fitted with a 4PL model by GraphPad Prism v.7 (GraphPad Software, San Diego, CA, USA) after luminescence readout with an EnVision 2104 Multilabel Reader (Perkin Elmer, Waltham, MA, USA).

## 5. Conclusions

Tryptophan is capable of degrading in cell culture media under elevated temperatures to afford colored and toxic degradation products. This is a concern for the development of room-temperature stable CCM, in which medium color change and a buildup of toxic compounds are undesirable. The storage of tryptophan-containing CCM at higher temperatures is best undertaken only if precautions are made to inhibit degradation into colored or toxic compounds. This study demonstrated that the addition of an antioxidant, namely alpha-ketoglutaric acid, is a viable means to achieve this goal of stabilizing CCM against problematic tryptophan degradation processes.

## Data Availability

Not applicable.

## Supplementary Materials

The following are available online at https://www.mdpi.com/article/10.3390/ijms22126221/s1.
