# Supplementary material for: Degradation Products of Tryptophan in Cell Culture Media: Contribution to Color and Toxicity
Source: Int J Mol Sci. 2021 Jun 9;22(12):6221. doi: 10.3390/ijms22126221 (PMC8228365; doi:10.3390/ijms22126221)
Supplement: Supplementary file 1 [file ijms-22-06221-s001.zip › ijms-1223841-supplementary.pdf]

# Degradation Products of Tryptophan in Cell Culture Media: Contribution to Color and Toxicity

Alisa Schnellbaecher, Anton Lindig, Maxime Le Mignon, Tim Hofmann, Brit Pardon, Stephanie Bellmaine, Aline Zimmer

## Supplementary Materials

### Section S1. CIELAB color analysis

L* represents the lightness (0 = opaque, 100 = transparent), a* represents the color in the green (negative a* value) to red (positive a* value) field and b* represents the color in the blue (negative b* value) to yellow (positive b* value) field. ΔE* represents the overall difference in color between two samples and is calculated using the formula (1)

$$\Delta E^{*} = \sqrt{\left(L^{*}{}_{2} - L^{*}{}_{1}\right)^{2} + \left(a^{*}{}_{2} - a^{*}{}_{1}\right)^{2} + \left(b^{*}{}_{2} - b^{*}{}_{1}\right)^{2}} \qquad (1)$$

### Section S2. LC-MS structure/tier assignments for features

Various levels of confidence were assigned to the annotations based on the initial recommendation of Sumner et al.[3] and some further published guidance document.[4] Briefly, tier 1 identification level was given to features where the retention time, the precursor mass and the fragmentation pattern matched a commercially available standard that was measured on the same LC-MS equipment and with the same method. Tier 2 annotations were obtained for features identified by comparing the MSMS fragmentation pattern with a spectral library (HMDB, METLIN or NIST). In a few cases, features were also annotated as tier 2 identification when the MSMS fragmentation pattern of the standard could not be confirmed using the experimental data (no MSMS) but for which the rt was correct (e.g. features of compounds **146**, **187**). Features were annotated as a tier 3 when a tentative structure matched the precursor mass and the MSMS fragmentation data could be annotated manually. Tier 4 correspond to features for which the precursor mass and the isotopic pattern were matched with a sum formula. Finally, Tier 5 were only identified by a unique retention time and a precursor mass and no tentative structure / sum formula can be reported.

### Section S3. Supplementary Figures

### Supporting Figure S1. Example of Tier 1 level identification

Experimental data obtained in the feed (blue) compared to the data obtained for the std (black) for feature m/z = 356.1021, rt = 10.30 min, tier 1, identified as compound **31**.

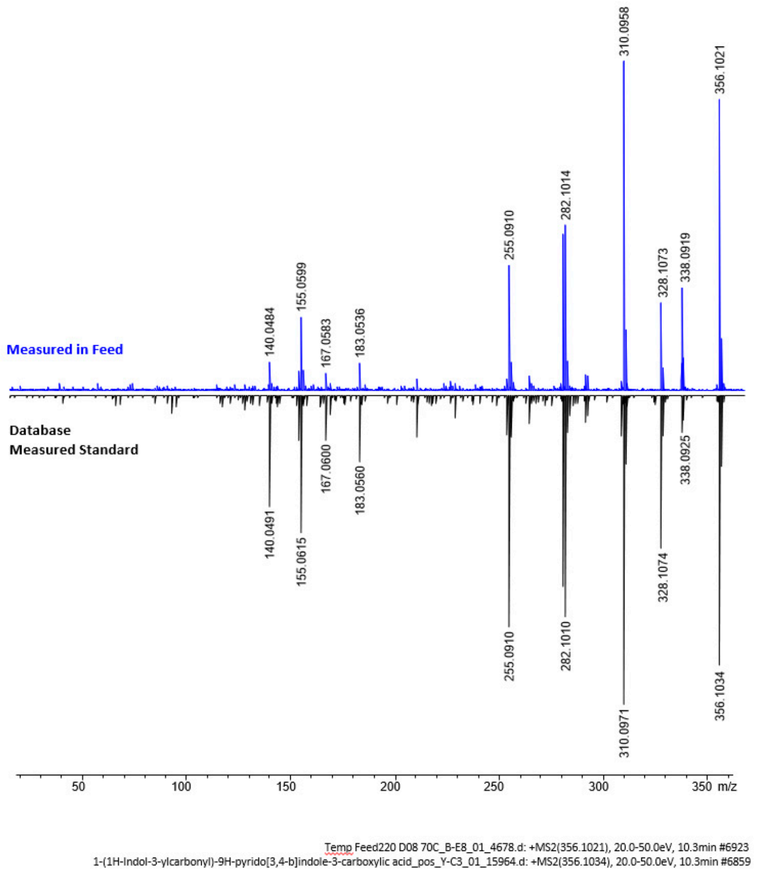

**Supplementary Figure S2. Heatmap of Tier 4-5 features that were significantly modulated in the aqueous Trp solutions.**

Row titles depict rt_m/z for each feature.

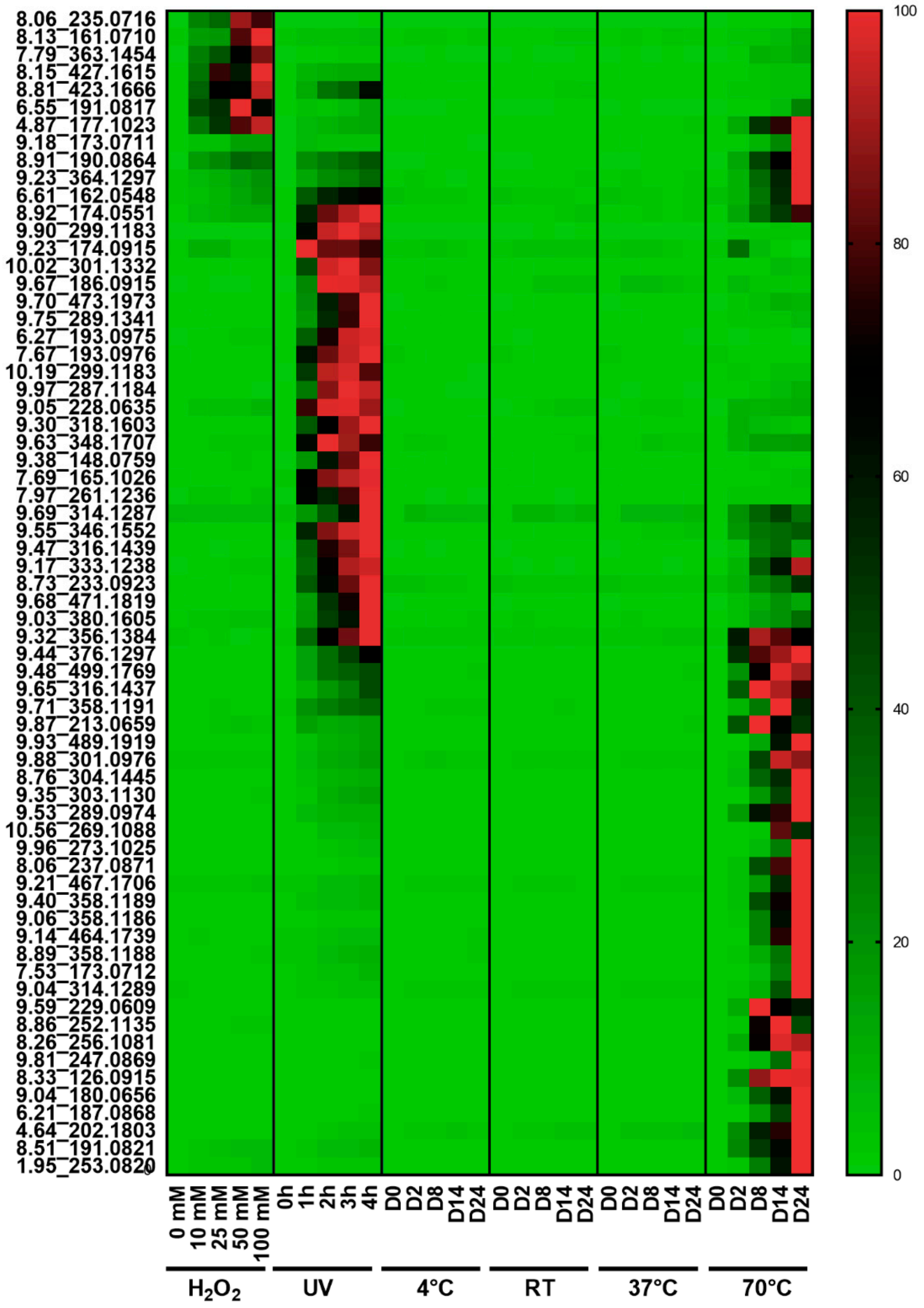

**Supplementary Table S1. LC-MS features identified in Trp-containing feed during 98-day storage at 37 °C.**

Features are exclusively those with abundance over 30,000 and which were not detected in feed-Trp. All m/z represent the parent peaks detected as $[M+H]^+$ ions, unless indicated otherwise. Asterisks next to the compound numbers indicate features which were also detected in the water study (Fig 4). The *q*-value was determined between the feed condition and the feed lacking Trp using the area under the curve of the abundance over time. n/a = not applicable.

| rt_m/z | Tier | Neutral Sum Formula | Compound # | Origin compound in feed (if known) | Abundance profile shape | Max. abundance | *q*-value |
|---|---|---|---|---|---|---|---|
| **6.76_205.0972** | 1 | C11H12N2O2 | Trp | NA | decreased | 4980057 | 2.41E-07 |
| **8.84_190.0864** | 4 | C11H11NO2 | n/a | Trp only | decreased | 2324057 | 0.00201 |
| **4.75_237.0871** | 1 | C11H12N2O4 | 4b* | Trp only | increased | 594623 | 1.55E-06 |
| **4.22_237.0871** | 1 | C11H12N2O4 | 4a* | Trp only | increased | 580469 | 5.09E-06 |
| **3.66_221.0922** | 2 | C11H12N2O3 | 7* | Trp only | increased | 360091 | 3.15E-06 |
| **9.06_146.0602** | 1 | C9H7NO | 18* | Trp only | increased | 302234 | 1.91E-05 |
| **7.73_146.0602** | 1 | C9H7NO | 20* | Trp only | increased | 284365 | 2.23E-06 |
| **10.24_356.1032** | 1 | C21H13N3O3 | 31* | Trp only | increased | 202762 | 2.54E-06 |
| **8.73_360.1343** | 3 | C21H17N3O3 | 30b* | Trp only | bell shape | 177370 | 2.96E-06 |
| **4.82_221.0922** | 1 | C11H12N2O3 | 6* | Trp only | increased | 140580 | 4.97E-06 |
| **5.43_209.0924** | 1 | C10H12N2O3 | 1* | Trp only | bell shape | 139838 | 1.23E-06 |
| **5.93_221.0922** | 1 | C11H12N2O3 | 3* | Trp only | decreased | 139749 | 0.00683 |
| **8.07_231.1129** | 1 | C13H14N2O2 | 22a* | Trp only | increased | 128150 | 2.12E-04 |
| **9.14_173.0714** | 4 | C10H8N2O | n/a | Trp only | bell shape | 115920 | 1.21E-06 |
| **6.21_175.0869** | 3 | C10H10N2O | 40 | Trp only | decreased | 83558 | 0.0117 |
| **8.75_183.0927** | 1 | C12H10N2 | 28* | Trp only | increased | 86875 | 3.81E-07 |
| **5.81_237.0871** | 1 | C11H12N2O4 | 2* | Trp only | decreased | 75753 | 7.51E-06 |
| **9.19_334.1549** | 1 | C20H19N3O2 | 14a* | Trp only | increased | 64468 | 1.99E-04 |
| **10.10_296.1184** | 3 | C20H13N3 | 36* | Trp only | increased | 59784 | 2.07E-05 |
| **8.61_360.1342** | 1* | C21H17N3O3 | 30a | Trp only | bell shape | 58239 | 9.52E-06 |
| **7.48_173.0711** | 4 | C10H8N2O | n/a | Trp only | increased | 57020 | 2.13E-04 |
| **9.76_312.1133** | 4 | C20H13N3O | n/a | Trp only | increased | 56607 | 9.30E-05 |
| **7.73_217.0975** | 1 | C12H12N2O2 | 22* | Trp only | increased | 52370 | 3.19E-06 |
| **9.37_314.1286** | 3 | C20H15N3O | 17* | Trp only | increased | 51898 | 9.64E-06 |
| **4.99_174.0551** | 1 | C10H7NO2 | 21* | Trp only | increased | 49542 | 2.50E-05 |
| **6.91_247.1078** | 1 | C13H14N2O3 | 25b* | Trp only | increased | 48912 | 1.29E-05 |
| **8.35_231.1129** | 1 | C13H14N2O2 | 23b* | Trp only | increased | 44544 | 1.16E-05 |
| **9.95_340.1083** | 1 | C21H13N3O2 | 38* | Trp only | increased | 44022 | 6.07E-06 |
| **9.29_173.0716** | 4 | C10H8N2O | n/a | Trp only | decreased | 37305 | 7.25E-04 |
| **9.05_366.1446** | 3 | C20H19N3O4 | 41 | Trp only | increased | 33732 | 2.60E-05 |
| **8.97_130.0650** | 2 | C9H7N | 8* | Trp only | increased | 31284 | 1.15E-05 |
| **8.53_288.1708**[a] | 3 | C16H22N3O2 | 43b | Choline | increased | 279132 | 1.77E-06 |
| **8.41_288.1708**[a] | 3 | C16H22N3O2 | 43a | Choline | increased | 142758 | 4.76E-06 |
| **5.88_304.1657**[a] | 3 | C16H22N3O3 | 44b | Choline | increased | 82782 | 7.51E-06 |
| **8.50_455.1859** | 5 | n/a | n/a | Thiamine | bell shape | 56144 | 4.30E-06 |
| **5.53_304.1657**[a] | 3 | C16H22N3O3 | 44a | Choline | increased | 32364 | 3.29E-05 |
| **8.25_354.145** | 1 | C19H19N3O4 | 42 | Pyridoxine | increased | 17201 | 1.29E-05 |
| **9.13_571.2122** | 5 | n/a | n/a | Thiamine | increased | 32235 | 1.12E-05 |
| **7.48_245.1284** | 4 | C14H16N2O2 | n/a | Leucine | increased | 399111 | 4.49E-06 |
| **9.13_289.1548** | 3 | C16H20N2O3 | 57 | Leucine | increased | 367727 | 3.61E-06 |

| | | | | | | | |
|---|---|---|---|---|---|---|---|
| **7.22_245.1284** | 4 | C14H16N2O2 | n/a | Isoleucine | increased | 297299 | 2.91E-05 |
| **7.19_348.1665** | 3 | C16H21N5O4 | 53b | Arginine | increased | 225147 | 1.67E-04 |
| **6.52_231.1128** | 4 | C13H14N2O2 | n/a | Arginine | increased | 191461 | 8.76E-07 |
| **6.94_245.1283** | 4 | C14H16N2O2 | n/a | Leucine | increased | 144996 | 6.75E-05 |
| **7.35_245.1286** | 4 | C14H16N2O2 | n/a | Isoleucine | increased | 139183 | 1.77E-05 |
| **8.26_289.1182** | 1* | C15H16N2O4 | 55 | Glutamic acid | increased | 101801 | 1.29E-05 |
| **6.18_348.1667** | 3 | C16H21N5O4 | 53a | Arginine | increased | 113533 | 1.05E-04 |
| **8.84_327.1089** | 3 | C16H14N4O4 | 48 | Histidine | increased | 95428 | 1.29E-05 |
| **6.70_187.1231** | 4 | C12H14N2 | n/a | Proline | increased | 87093 | 2.16E-05 |
| **5.06_295.1204** | 3 | C16H14N4O2 | 50 | Histidine | increased | 80139 | 7.89E-06 |
| **9.14_273.1600** | 1 | C16H20N2O2 | 56 | Leucine | increased | 67212 | 8.50E-06 |
| **8.32_314.1505** | 4 | C17H19N3O3 | n/a | Lysine | increased | 70476 | 4.38E-06 |
| **5.91_347.1350** | 3 | C16H18N4O5 | 49 | Histidine | plateau | 56107 | 2.12E-05 |
| **9.30_305.1490** | 3 | C16H20N2O4 | 59 | Leucine/Isoleucine | increased | 53700 | 0.0274 |
| **6.21_231.1128** | 4 | C13H14N2O2 | n/a | Arginine | increased | 52706 | 7.23E-06 |
| **7.09_316.1769** | 1 | C16H21N5O2 | 52 | Arginine | increased | 52599 | 6.69E-06 |
| **9.02_255.1132** | 4 | C15H14N2O2 | n/a | Lysine | increased | 36808 | 1.09E-05 |
| **8.63_275.1392** | 3 | C15H18N2O3 | 58 | Valine | increased | 44580 | 6.02E-06 |
| **6.93_302.1136** | 3 | C15H15N3O4 | 54 | Asparagine | increased | 43999 | 7.62E-06 |
| **8.45_291.0877** | 3 | C16H10N4O2 | 51 | Histidine | increased | 35000 | 8.12E-06 |
| **6.40_231.1128** | 4 | C13H14N2O2 | n/a | Arginine | increased | 36132 | 2.53E-05 |
| **5.17_313.1299** | 4 | C16H16N4O3 | n/a | Histidine | decreased | 32295 | 0.0403 |
| **8.26_219.1493** | 4 | C13H18N2O | n/a | Lysine | increased | 31203 | 2.56E-06 |
| **8.74_227.0816** | 1 | C13H10N2O2 | 45 | n/a | increased | 139010 | 1.09E-06 |
| **9.81_255.0765** | 1 | C14H10N2O3 | 46 | n/a | increased | 125241 | 9.28E-04 |
| **8.48_175.0867** | 2 | C10H10N2O | 47 | n/a | increased | 118659 | 5.90E-06 |
| **6.37_271.1078** | 4 | C15H14N2O3 | n/a | n/a | increased | 85358 | 2.17E-04 |
| **9.62_256.0718** | 4 | C13H9N3O3 | n/a | n/a | increased | 81088 | 2.52E-06 |
| **7.16_438.1595** | 5 | n/a | n/a | n/a | bell shape | 61133 | 2.49E-06 |
| **7.27_285.1234** | 4 | C16H16N2O3 | n/a | n/a | increased | 59718 | 1.06E-04 |
| **9.50_255.1133** | 4 | C15H14N2O2 | n/a | n/a | increased | 59709 | 6.75E-05 |
| **8.89_308.1394** | 4 | C18H17N3O2 | n/a | n/a | increased | 56190 | 3.72E-06 |
| **9.01_200.1285** | 4 | C10H17NO3 | n/a | n/a | increased | 54363 | 3.84E-05 |
| **9.61_416.1970** | 4 | C25H25N3O3 | n/a | n/a | increased | 52448 | 1.95E-05 |
| **9.36_402.1451** | 4 | C23H19N3O4 | n/a | n/a | increased | 52048 | 2.07E-05 |
| **6.15_321.0958**[b] | 4 | C15H14N4O3 | n/a | n/a | bell shaped | 50567 | 5.61E-05 |
| **9.43_307.0827** | 4 | C16H10N4O3 | n/a | n/a | increased | 40987 | 7.89E-06 |
| **8.81_292.1446** | 4 | C18H17N3O | n/a | n/a | bell shape | 40812 | 0.0154 |
| **8.92_321.1444** | 4 | C16H20N2O5 | n/a | n/a | increased | 39847 | 2.55E-04 |
| **8.20_263.0985** | 5 | n/a | n/a | n/a | increased | 38807 | 2.16E-05 |
| **8.74_321.1445** | 4 | C16H20N2O5 | n/a | n/a | increased | 38908 | 1.27E-04 |
| **7.72_333.1824** | 5 | n/a | n/a | n/a | decreased | 39198 | 5.78E-06 |
| **8.46_263.1391** | 4 | C14H19N2O3 | n/a | n/a | increased | 38908 | 6.21E-06 |
| **9.10_161.0720** | 4 | C9H8N2O | n/a | n/a | only D0 | 38607 | 0.00332 |
| **8.75_309.1234** | 4 | C18H16N2O3 | n/a | n/a | increased | 34524 | 1.39E-05 |

a. m/z = $[M]^+$
b. m/z = $[M+Na]^+$

## Supplementary Table S2. CHO cell toxicity assay

All compounds were tested in the CHOK1 GS cell line, and those which showed toxicity in that cell line were tested in the other three cell lines also. n/a = not applicable, as compound was not tested.

| Compound # | $[GI_{50}]$ (μM) | | | |
|---|---|---|---|---|
| | CHOK1 GS | CHOK1 | CHOZN 6 | CHOZN 10 |
| **39** | < 0.13 | 0.2 | 1.1 | 1.05 |
| **34** | 24.0 | 10.6 | 12.3 | 13.1 |
| **31** | 34.6 | 21.6 | 13.0 | 14.4 |
| **12** | 64.5 | 115.8 | 54.5 | 92.3 |
| **16** | 76.6 | 74.9 | 88.5 | 57.6 |
| **5** | 118.2 | 158.0 | 76.7 | 75.5 |
| **38** | 118.4 | 123.5 | 98.9 | 114.0 |
| **27** | 127.4 | 74.8 | 98.5 | 64.5 |
| **29** | 156.7 | 388.8 | >1000 | 347.4 |
| **28** | 170 | 74.9 | 65.5 | 77.3 |
| **18** | 295.4 | 226.4 | 236.0 | 257.5 |
| **24** | 342.7 | 311.9 | 314.4 | 306.0 |
| **1** | >1000 | n/a | n/a | n/a |
| **2** | >1000 | n/a | n/a | n/a |
| **3** | >1000 | n/a | n/a | n/a |
| **4** | >1000 | n/a | n/a | n/a |
| **6** | >1000 | n/a | n/a | n/a |
| **8** | >1000 | n/a | n/a | n/a |
| **9** | >1000 | n/a | n/a | n/a |
| **10** | >1000 | n/a | n/a | n/a |
| **11** | >1000 | n/a | n/a | n/a |
| **14** | >1000 | n/a | n/a | n/a |
| **19** | >1000 | n/a | n/a | n/a |
| **20** | >1000 | n/a | n/a | n/a |
| **21** | >1000 | n/a | n/a | n/a |
| **22** | >1000 | n/a | n/a | n/a |
| **23** | >1000 | n/a | n/a | n/a |
| **25** | >1000 | n/a | n/a | n/a |
| **32** | >1000 | n/a | n/a | n/a |
| **42** | >1000 | n/a | n/a | n/a |
| **45** | >1000 | n/a | n/a | n/a |
| **46** | >1000 | n/a | n/a | n/a |
| **47** | >1000 | n/a | n/a | n/a |
| **56** | >1000 | n/a | n/a | n/a |
| **60** | >1000 | n/a | n/a | n/a |
| **62** | >1000 | n/a | n/a | n/a |

**Supplementary Figure S3. Heatmap of Tier 1 to 3 features in the feed, feed-Trp and feed+aKG solutions stored at 37°C.**

Row titles depict rt_m/z for each feature.

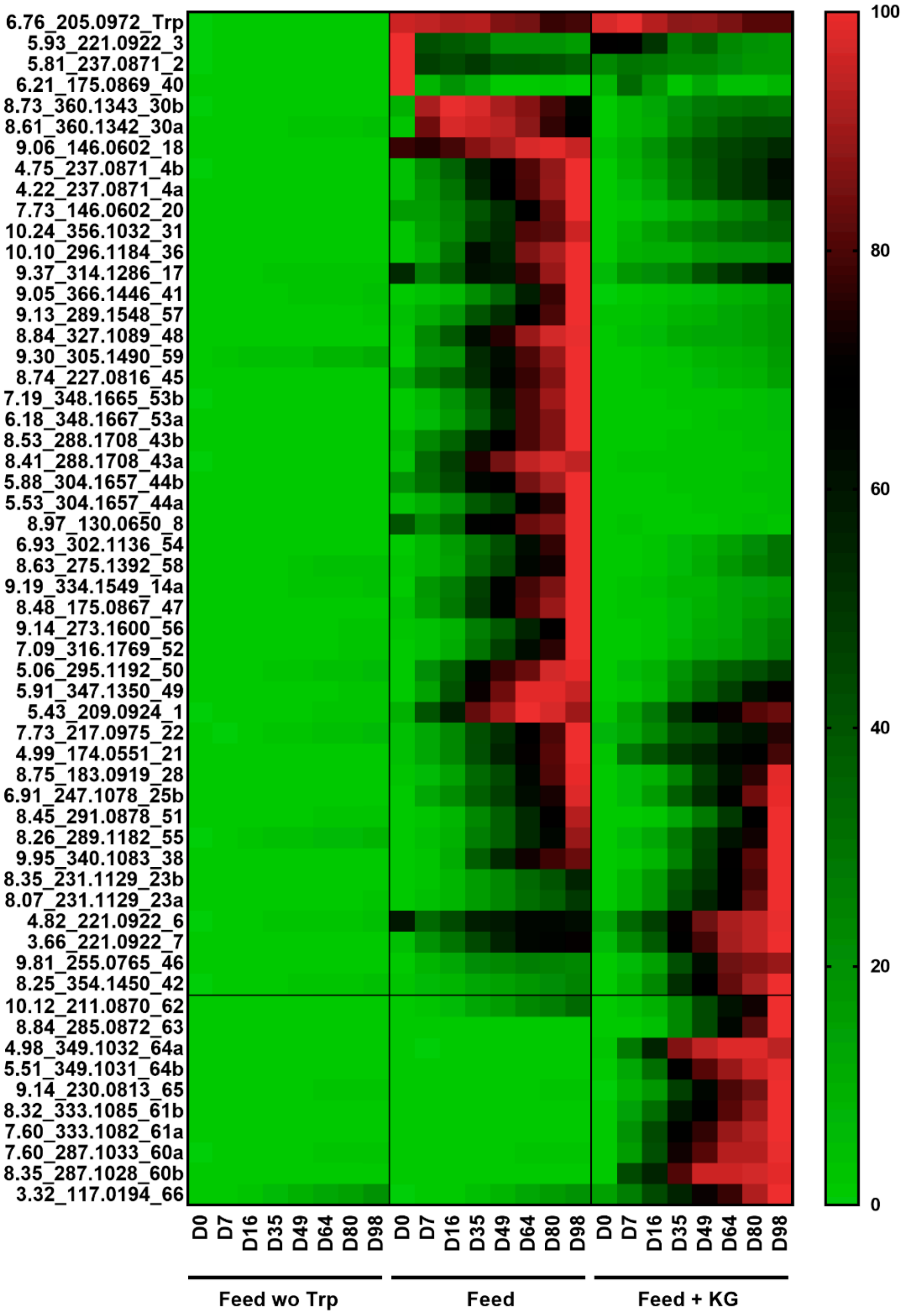

**Supplementary Figure S4. Heatmap of Tier 4 and 5 features in the feed, feed-Trp and feed+aKG solutions stored at 37°C.**

Row titles depict rt_m/z for each feature.

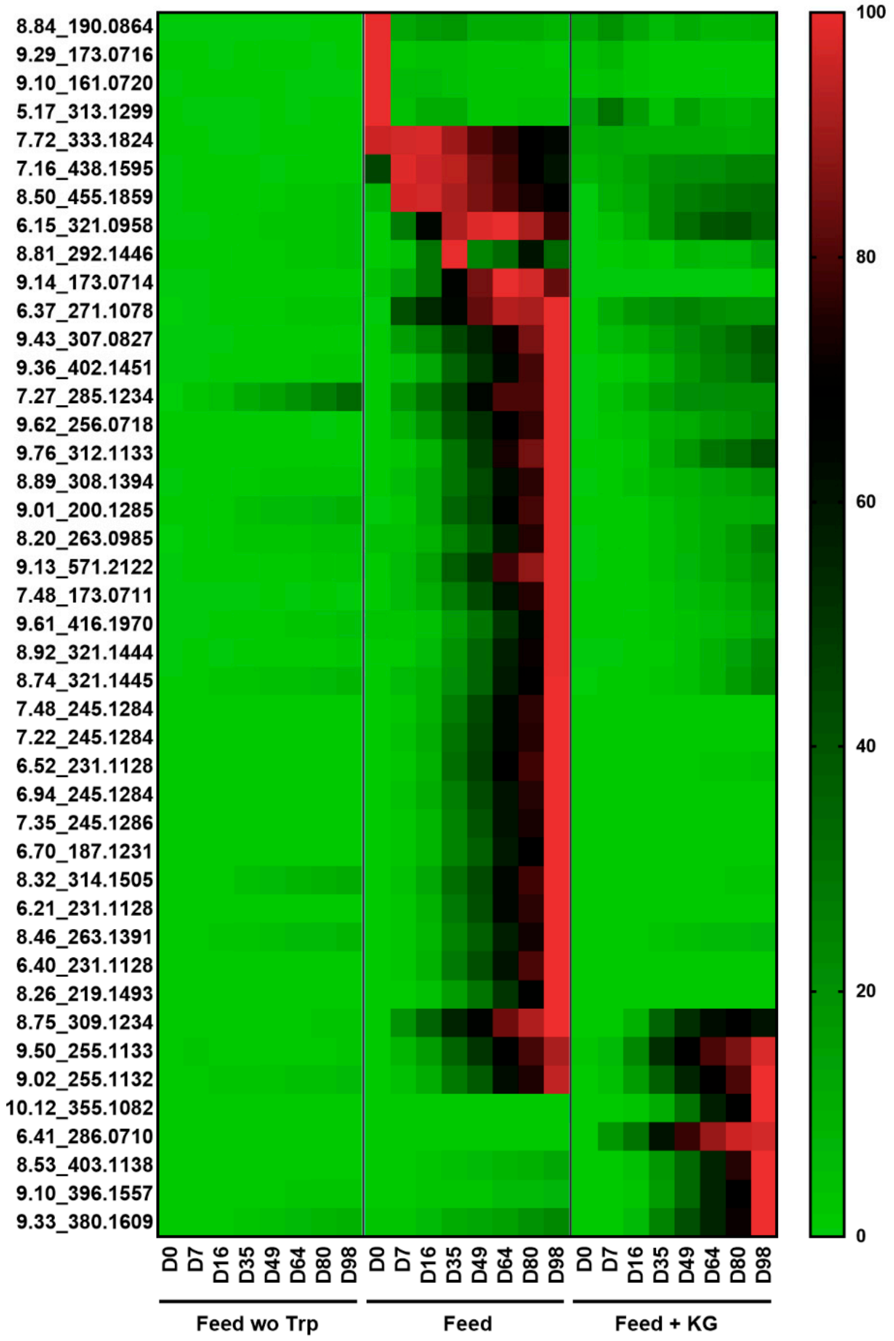

**Supplementary Table S3. Commercial origin of compounds used as standards for Tier 1-2 feature identification.**

| Compound name | CAS | Compound # | Catalogue # | Supplier |
|---|---|---|---|---|
| L-Tryptophan | 200-795-6 | **-** | T0254 | Sigma |
| N-Formylkynurenine | 3978-11-8 | **2** | T-205 | TLC Pharmaceutical Standards |
| Kynurenine | 343-65-7 | **1** | 61250 | Sigma |
| Indole-3-acetaldehyde | 2591-98-2 | **12** | BN22755 | Aurum Pharmatech |
| Indole-3-carboxaldehyde | 487-89-8 | **18** | 129445 | Sigma |
| 2-amino-3-(2-oxo-2,3-dihydro-1h-indol-3-yl)propanic acid (Oia) | 21704-80-3 | **3** | FCH3581490 | ChemSpace |
| 3-(3-Hydroxy-2-oxo-2,3-dihydro-1H-indol-3-yl)-L-alanine (diOia) | 184955-21-3 | **4** | T-204 | TLC Pharmaceutical Standards |
| 1,2,3,3a,8,8a-Hexahydro-3a-hydroxypyrrolo[2,3-b]indole-2-carboxylic acid (PIC) | 35169-97-2 | **6** | CB0162344 | Chemieliva |
| 2,3,4,9-Tetrahydro-1H-beta-carboline-1-carboxylic acid | 6052-68-2 | **22** | S277886 | Sigma |
| 1-Methyl-2,3,4,9-tetrahydro-1H-beta-carboline-3-carboxylic acid | 5470-37-1 | **23** | H0500 | Sigma |
| 2-(3-Indolylmethyl)-L-tryptophan | 149724-31-2 | **14** | T-2014 | TLC Pharmaceutical Standards |
| Tryptamine | 61-54-1 | **11** | 193747 | Sigma |
| 4-Hydroxyquinoline | 611-36-9 | **20** | H58005 | Sigma |
| Norharmane | 244-63-3 | **27** | N6252 | Sigma |
| Harmane | 486-84-0 | **28** | 103276 | Sigma |
| 1-Acetyl-β-carboline | 50892-83-6 | **62** | AG-B09388 | Angel Pharmatech |
| 1H-Pyrido[3,4-b]indole-1,3-dicarboxylic acid, 1-(2-carboxyethyl)-2,3,4,9-tetrahydro-, (1S-cis)- | 122110-59-2 | **60** | CE1002256 | Chemieliva |
| 2-Oxindole | 59-48-3 | **19** | O9808 | Sigma |
| 1-(1H-Indol-3-ylcarbonyl)-9H-pyrido[3,4-b]indole-3-carboxylic acid | 863766-95-4 | **31** | CC0472247 | Chemieliva |
| 9H-Pyrido[3,4-b]indole-1-carboxylic acid | 26052-96-0 | **5** | EN300-205447 | Enamine |
| 1-(1H-Indol-3-yl)-9H-pyrido[3,4-b]indole | 155885-64-6 | **34** | 842D79 | WuXi AppTec |
| 3-Indoleacetamide | 879-37-8 | **47** | 286281 | Sigma |
| Quinoline | 91-22-5 | **8** | 241571 | Sigma |
| 1H-Indol-3-yl-9H-pyrido[3,4-b]indol-1-ylmethanone | 244295-64-5 | **32** | 9FE868 | WuXi AppTec |
| 2,3,4,9-Tetrahydro-1-(2-methylpropyl)-1H-pyrido[3,4-b]indole-3-carboxylic acid | 146436-31-9 | **56** | Amb17872939 | Ambinter |
| 4-Quinolinecarboxylic acid | 486-74-8 | **21** | 174823 | Sigma |

| | | | | |
|---|---|---|---|---|
| 1-methyl-9H-pyrido[3,4-b]indole-3-carboxylic acid | 22329-38-0 | **45** | MSC1806155A | Merck Serono Research |
| 4,9-dihydro-3H-pyrido[3,4-b]indole-3-carboxylic acid | 46501-39-7 | **24** | CSC009997543 | Chemspace |
| 1-Phenyl-9H-pyrido[3,4-b]indole-3-carboxylic acid | 374710-96-0 | **16** | LN00360728 | Labnetwork |
| 1-Acetyl-3-carboxy-β-carboline | 73818-29-8 | **46** | CB0358346 | Chemieliva |
| 1H-Pyrido[3,4-b]indole-3-carboxylic acid, 2,3,4,9-tetrahydro-1-[3-hydroxy-5-(hydroxymethyl)-2-methyl-4-pyridinyl]- | 158249-61-7 | **42** | 0DE6DA | Intonation Research Laboratories |
| 2-Hydroxytryptamine | 13078-93-8 | **9** | CA0206490 | Chemieliva |
| Tryptanthrin | 13220-57-0 | **39** | SML0310 | Sigma |
| 9H-Pyrido[3,4-b]indole-1-methanol | 17337-22-3 | **29** | CA0354102 | Chemieliva |
| 1H,2H,3H,3aH,8H,8aH-Pyrrolo[2,3-b]indol-3a-ol | 58635-39-5 | **10** | CA0659218 | Chemieliva |
| 2-[(E)-ethylideneamino]-3-(2-oxo-2,3-dihydro-1H-indol-3-yl)propanoic acid | No CAS | **25b** | 5A768B | WuXi AppTec |

## Section S4. Synthesis of standards for LC-MS

All reagents were used as received from commercial suppliers. All NMR spectra were recorded at 298K on a 700MHz Bruker Advance III spectrometer equipped with a cryo cooled TCI probe. Chemical shifts were reported in parts per million (ppm), and the residual solvent peak was used as an internal reference: proton (chloroform δ 7.26, DMSO δ 2.50), carbon (chloroform δ 77.16, DMSO δ 39.52). Multiplicity was indicated as follows: s (singlet), d (doublet), t (triplet), q (quartet), m (multiplet), dd (doublet of doublet). HRMS was obtained using the Impact II mass spectrometer equipped with an ESI source (Bruker Daltonics, Bremen, Germany).

**1-(1*H*-indole-3-carbonyl)-1,2,3,4-tetrahydro-β-carboline-3-carboxylic acid (30)** Tetrahydro-β-carboline **30** was synthesized according to literature protocol,[5] and as per the literature precedent, was too unstable to allow NMR characterization. HRMS (ESI): m/z calcd for $C_{21}H_{18}N_3O_3+H^+$: 360.1343 $[M+H]^+$ 360.1340.

**1-(Indol-3-yl)-β-carboline-3-carboxylic acid (33)** β-Carboline **33** was synthesized according to literature protocol.[6] 1H NMR(700 MHz, $d_6$-DMSO) δ = 12.04 (2H, bs, NH), 8.87 (1H, s, Ar-H), 8.67 (1H, bs, Ar-H), 8.51 (1H, m, Ar-H), 8.44 (1H, m, Ar-H), 7.80 (1H, m, Ar-H), 7.64 (1H, m, Ar-H), 7.57 (1H, m, Ar-H), 7.36 (1H, m, Ar-H), 7.27 (1H, m, Ar-H), 7.21 (1H, m, Ar-H); 13C NMR (176 MHz, DMSO-$d_6$) δ = 166.25, 141.78, 139.11, 136.57, 135.19, 133.30, 128.97, 128.80, 127.73, 126.13, 122.47, 122.10, 122.03, 121.42, 120.69, 120.43, 114.68, 113.01, 111.82, 110.82; HRMS (ESI): m/z calcd for $C_{20}H_{14}N_3O_2+H^+$: 328.1081 $[M+H]^+$ 328.1081.

**1-(Quinolin-4-yl)-β-carboline-3-carboxylic acid (38)** 4-Quinonlinecarboxaldehyde (150 mg, 0.95 mmol) was added to a suspension of L-tryptophan (195 mg, 0.95 mmol) in ethanol (8 ml). Trifluoroacetic acid (0.80 ml, 10.38 mmol) was added and the solution was stirred for 6 days under nitrogen at room temperature, during which a precipitate formed. The precipitate was filtered and washed with ethyl acetate to afford the β-carboline **38** as a yellow-orange powder (100 mg, 31%). 1H NMR (400 MHz, $d_6$-DMSO) δ = 11.75 (1H, s, NH), 9.21 (1H, m, Ar-H), 9.08 (1H, s, Ar-H), 8.47 (1H, m, Ar-H), 8.24 (1H, m, Ar-H), 7.93-7.88 (2H, m, Ar-H), 7.75 (1H, m, Ar-H), 7.63-7.53 (3H, m, Ar-H), 7.36 (1H, m, Ar-H); 13C NMR (176 MHz, DMSO-$d_6$) δ = 166.68, 149.29, 141.52, 138.77, 137.35, 136.00, 130.82, 129.46, 129.11, 127.99, 127.89, 127.71, 126.29, 126.04, 122.52, 122.39, 121.07,

120.60, 120.43, 117.70, 112.53; HRMS (ESI): m/z calcd for $C_{21}H_{14}N_3O_4$ +$H^+$: 340.1081 $[M+H]^+$ 340.1080.

**2-($N^{\tau}$-histidino)-4-(2-aminophenyl)-4-oxobutanoic acid (KYN-His adduct, 49)** KYN-His adduct **49** was synthesized according to literature protocol,[7] and as per the literature precedent, was too unstable to allow NMR characterization. HRMS (ESI): m/z calcd for $C_{16}H_{18}N_4O_5$ +$H^+$: 347.1350 $[M+H]^+$ 347.1353.

1-(3-Guanidinopropyl)-1,2,3,4-tetrahydro-*b*-carboline-3-carboxylic acid (**52**) was synthesized in two steps from L-tryptophan methyl ester.

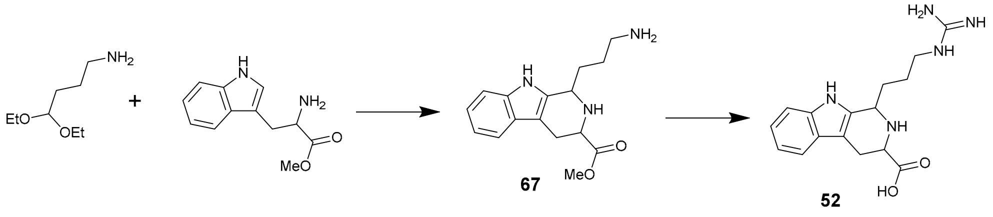


**Methyl 1-(3′-aminopropyl)-1,2,3,4-tetrahydro-β-carboline-3-carboxylate (67)** A solution of 4-aminobutyraldehyde diethyl acetal (90%, 0.21 ml, 1.12 mmol) in dichloromethane (2 ml) was cooled to 0 °C. Trifluoroacetic acid (1 ml, 13 mmol) and water (1 ml) were added and the solution was stirred at 0 °C for 45 min. Tryptophan methyl ester (284.4 mg, 1.12 mmol) was added and the solution was allowed to warm to room temperature and stirred overnight. The solution was concentrated under reduced pressure and purified using flash chromatography (0-50% MeOH/EtOAc + 2% $NH_4OH$) to afford the tetrahydro-β-carboline **67** as a yellow oil (303 mg, 94%). $\delta_H$ (400 MHz, $d_6$-DMSO) 11.53, (1H, s, NH), 8.26 (2H, bs, NH2), 7.48 (1H, m, Ar-H), 7.38 (1H, m, Ar-H), 7.12 (1H, m, Ar-H), 7.02 (1H, m, Ar-H), 4.79 (1H, m, H1), 4.52 (1H, m, H3), 3.85 (3H, s, CH3), 3.30-3.10 (2H, m, H4), 2.91 (2H, m, H3′), 2.49-2.2.14 (2H, m, H2′), 2.03-1.80 (2H, m, H1′); 13C NMR (101 MHz, DMSO-d6) δ = 168.87, 136.46, 129.67, 125.52, 121.81, 119.11, 118.00, 111.44, 104.93, 55.05, 52.93, 52.78, 38.17, 27.80, 22.50, 22.19; HRMS (ESI): m/z calcd for $C_{16}H_{22}N_3O_2$ +$H^+$: 288.1707 $[M+H]^+$ 288.1701.

**1-(3′-Guanidinopropyl)-1,2,3,4-tetrahydro-β-carboline-3-carboxylic acid (52)** S-methylisothiouronium sulfate (1.45 g, 5.22 mmol) was added to a solution of NaOH(aq.) (1M, 5.2 ml, 5.22 mmol) in methanol (18 ml) at 0°C. The solution was stirred at 0°C for 5 min, upon which a precipitate formed. The precipitate was filtered off, and amine **67** (2.00 g, 6.96 mmol) was added to the filtrate. The solution was stirred at room temperature for 3 days. A solution of 25% ammonia (aq.) (4 ml) was added to the reaction mixture, which was then stirred at reflux for 2 h. The solution was concentrated by half using a rotary evaporator and then brought to pH 7 with aqueous HCl (10M), upon which a precipitate formed. The precipitate was filtered and triturated with hot methanol to afford carboxylic acid **52** as a white solid (88 mg, 4%) 1H NMR (400 MHz, $d_6$-DMSO) δ = 11.24 (1H, s, NH), 8.60 (1H, bs, NH), 7.60 (3H, bs, guanidino), 7.42 (1H, m, Ar-H), 7.36 (1H, m, Ar-H), 7.08 (1H, m, Ar-H),, 6.99 (1H, m, Ar-H), 4.60 (1H, m, H1), 3.78 (1H, m, H3), 3.21-2.83 (2H, m, H4), 3.20 (2H, m, H3′), 2.32-2.04 (2H, m, H2′), 1.87-1.62 (2H, m, H1′); 13C NMR (101 MHz, DMSO-d6) δ = 171.84, 157.30, 136.52, 131.25, 126.01, 121.36, 118.81, 117.88, 111.35, 106.85, 57.47, 52.59, 40.50, 28.63, 23.82, 23.23; HRMS (ESI): m/z calcd for $C_{16}H_{22}N_5O_2$ +$H^+$: 316.1768 $[M+H]^+$ 316.1763.

1-(2-Carboxyethyl)-1,2,3,4-tetrahydro-β-carboline-3-carboxylic acid (**55**) was synthesized in two steps from L-tryptophan methyl ester.

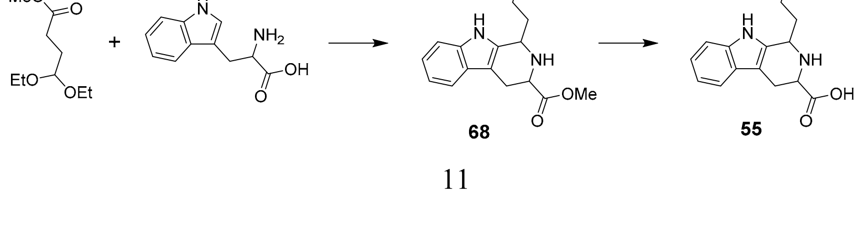

**Methyl (1-(3-Methyl propanoate)-1,2,3,4-tetrahydro-β-carboline)-3-carboxylate (68)** A solution of methyl 4,4-dimethoxybutyrate (200 mg, 1.2 mmol) in DCM (2 ml) was cooled to 0 °C. TFA (1 ml, 13.0 mmol) and water (1 ml) were added to the solution, and the solution was stirred at 0 °C for 45 min. L-Tryptophan methyl ester (305 mg, 1.2 mmol) was added to the solution, then the solution was allowed to warm to room temperature and stirred overnight. The reaction mixture was concentrated under reduced pressure and purified using column chromatography (10-100% MeOH/EtOAc) to afford dimethyl ester **68** as a pale yellow oil (154 mg, 41%). 1H NMR (400 MHz, $d_6$-DMSO) δ = 11.26 (1H, s, NH), 7.50 (1H, m, Ar-H), 7.39 (1H, m, Ar-H), 7.14 (1H, m, Ar-H), 7.04 (1H, m, Ar-H), 4.80 (1H, m, H1), 4.61-4.57 (1H, m, H3), 3.87 (3H, s, CH3), 3.66 (3H, s, CH3), 3.33-3.01 (2H, m, H4), 2.73-2.56 (2H, m, CH2), 2.62-2.18 (2H, m, CH2). 13C NMR (101 MHz, DMSO-$d_6$) δ = 172.52, 169.15, 136.54, 129.33, 125.55, 122.00, 119.25, 118.08, 111.50, 105.15, 55.09, 53.11, 53.06, 51.54, 28.86, 25.95, 22.38. HRMS (ESI): m/z calcd for $C_{17}H_{20}N_2O_4$+$H^+$: 317.1496 $[M+H]^+$ 317.1495.

**1-(2′-Carboxyethyl)-1,2,3,4-tetrahydro-β-carboline-3-carboxylic acid (55)** LiOH (238 mg, 9.7 mmol) was added to a solution of methyl carboxylate **68** (154 mg, 0.5 mmol) in 3:1 THF/water (16 ml) and the solution was stirred at reflux overnight. The solution was allowed to cool to room temperature and was neutralized using Amberlite IR120 H+ ion exchange resin. The solution was concentrated under reduced pressure to afford carboxylic acid **55** as pale yellow, waxy oil (142 mg, quant.). 1H NMR (400 MHz, $d_6$-DMSO) δ = 11.05 (1H, s, NH), 7.36 (1H, m, Ar-H), 7.30 (1H, m, Ar-H), 7.02 (1H, m, Ar-H), 6.94 (1H, m, Ar-H), 4.34 (1H, m, H1), 3.89 (1H, m, H3), 3.05-2.62 (2H, m, H4), 2.34-1.93 (4H, m, 2 × CH2). HRMS (ESI): m/z calcd for $C_{15}H_{16}N_2O_4$+$H^+$: 289.1183 $[M+H]^+$ 289.1179.

## Section S5. References


[1] C. A. Smith, G. O'Maille, E. J. Want, C. Qin, S. A. Trauger, T. R. Brandon, D. E. Custodio, R. Abagyan, G. Siuzdak, *Ther. Drug Monit.* **2005**, *27*, 747-751.

[2] C. Guijas, J. R. Montenegro-Burke, X. Domingo-Almenara, A. Palermo, B. Warth, G. Hermann, G. Koellensperger, T. Huan, W. Uritboonthai, A. E. Aisporna, D. W. Wolan, M. E. Spilker, H. P. Benton, G. Siuzdak, *Analytical Chemistry* **2018**, *90*, 3156-3164.

[3] L. W. Sumner, A. Amberg, D. Barrett, M. H. Beale, R. Beger, C. A. Daykin, T. W. Fan, O. Fiehn, R. Goodacre, J. L. Griffin, T. Hankemeier, N. Hardy, J. Harnly, R. Higashi, J. Kopka, A. N. Lane, J. C. Lindon, P. Marriott, A. W. Nicholls, M. D. Reily, J. J. Thaden, M. R. Viant, *Metabolomics* **2007**, *3*, 211-221.

[4] I. Blaženović, T. Kind, M. R. Sa, J. Ji, A. Vaniya, B. Wancewicz, B. S. Roberts, H. Torbašinović, T. Lee, S. S. Mehta, M. R. Showalter, H. Song, J. Kwok, D. Jahn, J. Kim, O. Fiehn, *Analytical Chemistry* **2019**, *91*, 2155-2162.

[5] P. Zhang, X. Sun, B. Xu, K. Bijian, S. Wan, G. Li, M. Alaoui-Jamali, T. Jiang, *European Journal of Medicinal Chemistry* **2011**, *46*, 6089-6097.

[6] L. P. P. Liew, J. M. Fleming, A. Longeon, E. Mouray, I. Florent, M.-L. Bourguet-Kondracki, B. R. Copp, *Tetrahedron* **2014**, *70*, 4910-4920.

[7] S. Vazquez, J. A. Aquilina, J. F. Jamie, M. M. Sheil, R. J. W. Truscott, *Journal of Biological Chemistry* **2002**, *277*, 4867-4873.
